# Relationship between occupational stress and job burnout among rural-to-urban migrant workers in Dongguan, China: a cross-sectional study

**DOI:** 10.1136/bmjopen-2016-012597

**Published:** 2016-08-17

**Authors:** Hao Luo, Hui Yang, Xiujuan Xu, Lin Yun, Ruoling Chen, Yuting Chen, Longmei Xu, Jiaxian Liu, Linhua Liu, Hairong Liang, Yali Zhuang, Liecheng Hong, Ling Chen, Jinping Yang, Huanwen Tang

**Affiliations:** 1 Department of Environmental and Occupational Health, Dongguan Key Laboratory of Environmental Medicine, School of Public Health, Guangdong Medical University, Dongguan, People's Republic of China; 2 Post Graduate Academic Institute of Medicine, and Faculty of Education, Health and Wellbeing, University of Wolverhampton, Wolverhampton, UK; 3 Baoan Center for Disease Control and Prevention of Shenzhen, Shenzhen, People's Republic of China

**Keywords:** occupational stress, job burnout, Rural-to-urban Migrant Workers

## Abstract

**Objectives:**

In China, there have been an increasing number of migrant workers from rural to urban areas, and migrant workers have the highest incidence of occupational diseases. However, few studies have examined the impact of occupational stress on job burnout in these migrant workers. This study aimed to investigate the relationship between occupational stress and job burnout among migrant workers.

**Design:**

This study used a cross-sectional survey.

**Setting:**

This investigation was conducted in Dongguan city, Guangdong Province, China.

**Participants:**

3806 migrant workers, aged 18–60 years, were randomly selected using multistage sampling procedures.

**Primary and secondary outcome measures:**

Multistage sampling procedures were used to examine demographic characteristics, behaviour customs and job-related data. Hierarchical linear regression and logistic regression models were constructed to explore the relationship between occupational stress and burnout.

**Results:**

Demographics, behaviour customs and job-related characteristics significantly affected on burnout. After adjusting for the control variable, a high level of emotional exhaustion was associated with high role overload, high role insufficiency, high role boundary, high physical environment, high psychological strain, high physical strain, low role ambiguity, low responsibility and low vocational strain. A high level of depersonalisation was associated with high role overload, high role ambiguity, high role boundary, high interpersonal strain, high recreation, low physical environment and low social support. A low level of personal accomplishment was associated with high role boundary, high role insufficiency, low responsibility, low social support, low physical environment, low self-care and low interpersonal strain. Compared to the personal resources, the job strain and personal strain were more likely to explain the burnout of rural-to-urban migrant workers in our study.

**Conclusions:**

The migrant workers have increased job burnouts in relation to occupational stress. Relieving occupational stress and maintaining an appropriate quantity and quality of work could be important measures for preventing job burnout among these workers.

**Strengths and limitations of this study**

- The current study is the first to examine the relationship between occupational stress and job burnout among rural-to-urban migrant workers in China.
- However, this study is a cross-sectional design, being cautious for the causal relationship between occupational stress and job burnout. Owing to self-report measures, there may be an inherent bias in the data collection.
- Multistage, stratified random sampling design was used in this study to make the data representative.

## Introduction

Stress is an important element of life, and appropriate levels of stress can help an individual overcome challenging situations. However, if not managed well, high levels of stress will result in emotional problems and ill health.^1^ Stress-related symptoms range from mild medical unfitness, general unhappiness and anxiety to more serious conditions, including drug dependency, excessive drinking, increased smoking, divorce, psychiatric problems and suicide.^2^ Stress has been found to be strongly associated with job satisfaction; increased levels of stress can lead to reduced job satisfaction. Occupational stress is defined as harmful physical and emotional responses incurred in the work environment. As jobs have shifted from manufacturing industries to service industries, the psychological and emotional demands of work have increased. This has led to an increased awareness of work-related burnout.^3^

Burnout is described as a prolonged response to chronic physical, emotional and mental exhaustion at work, which is characterised by emotional exhaustion (EE), depersonalisation (DEP) and reduced personal accomplishment (PA).^4^ ^5^ It has been recognised as an occupational hazard and is associated with physical illness and mental problems, including cardiovascular disease, musculoskeletal pain, depression and anxiety.^6^ ^7^ Additionally, burnout is associated with absenteeism, intention to leave the job and actual turnover, and lower productivity, job performance and involvement.^7–9^

Rural-to-urban migrant workers, who migrate from the rural areas of their original residence to urban areas, are a unique phenomenon occurring in low and middle income countries (LMC) with experiencing economic transformations. China is the largest population LMC, and over the past three decades, it has had a rapid economic development, with having produced the largest human migration in history. By 2012, the number of migrant workers had reached 263 million.^10^ Most migrant workers gathered in economically developed areas such as the Pearl River Delta in Guangdong Province. Dongguan lies in the Pearl River Delta, and many of its small and medium-sized enterprises were assembled by migrant workers. Although migrant workers have become a vital labour supply to Dongguan's economy, this population lacks health and safety protection. Previous studies showed that because of their exposure to poor working conditions, occupational hazards and long working hours,^11^ ^12^ migrant workers have suffered from the highest incidences of occupational diseases in all labour force in China.^13^ Although working environments have improved with modernisation, higher levels of knowledge and productivity are now being demanded from migrant workers. Under these circumstances, the degree of acceptance felt in a new home, relationships with coworkers, job-related and behaviour customs, interpersonal tensions and conflicts may lead to occupational stress for migrant workers. Meanwhile, evidence from earlier studies suggests a positive association between the employees' health and burnout,^14^ ^15^ whereas demographics and job-related characteristics may also be important factors influencing the level of burnout.^15^ ^16^ Additionally, occupational stress has been strongly associated with burnout in medical and nursing populations.^17^ ^18^ However, there are few studies on the relationship between occupational stress and burnout among rural-to-urban migrant workers. Since this population is unique and its numbers are increasing, it is necessary to investigate the relationships between occupational stress and burnout. We carried out a cross-sectional study among rural-to-urban migrant workers in Dongguan to investigate (1) the relationship between migration characteristics and burnout among rural-to-urban migrant workers and (2) the association between occupational stress and burnout. The results could help deepen our understanding of the factors associated with burnout and underscore the need to establish policies to protect rural-to-urban migrant workers' health.

## Methods

### Study design and participants

This cross-sectional survey was performed among migrant workers from the Guancheng District of Dongguan, China. The participants in this study were recruited using a multistage, stratified sampling method. In the first stage, two towns were randomly selected from eight towns in Guancheng based on economic considerations and the proportion of migrants in these towns. In the second stage, we randomly selected factories in electronics, shoe making, the chemical industry and furniture by applying computer-generated random numbers to the town's list of factories. In the third stage, workers were randomly selected for participation from the sampled factories, which employed ∼10 000 rural-to-urban migrant workers. All participants were informed about the study and were invited to fill out an anonymous self-administered questionnaire between March 2013 and May 2013. The inclusion criteria for participation were as follows: (1) aged 18–60 years; (2) without a local household registration in Dongguan; (3) having left their original home and resided in Dongguan for at least 6 months and (4) willingness to provide verbal informed consent. The exclusion criteria were individuals who might have difficulties in understanding and answering the questionnaire, even with the help of facilitators. Initially, there were 4500 rural-to-urban migrant workers who were available and who met the criteria. Formal consent was given by 4463 respondents (99.17%) who completed the questionnaires. A total of 657 cases with missing data were excluded from the 4463 acquired data sets, resulting in a total of 3806 questionnaire data sets used in the final analysis. The overall complete response rate was 84.58%. This study was obtained under a protocol approved by the Guangdong Medical University Ethics and Human Subjects Committee. Written informed consent was obtained from all study participants.

### Measurement of occupational stress

Occupational stress was assessed using the Occupational Stress Inventory—revised edition (OSI-R) written by Osipow^19^ and adapted by Li. The scale consists of the following three dimensions: the Occupational Role Questionnaire (ORQ) (60 items), the Personal Strain Questionnaire (PSQ) (40 items) and the Personal Resources Questionnaire (PRQ) (40 items).The respondent scores the frequency of a particular behaviour on a Likert scale from 1 (never) to 5 (often).^20^ For the ORQ and PSQ, a higher score indicates more nervousness. A higher score for the PRQ indicates that the respondent has a stronger ability to cope with tension. In each dimension, we divided the participants into three parts according to tertiles of their scores as low, moderate and high score groups. The following were the cut-off points for the OSI-R subscales—ORQ: low<120, moderate 120–160, high >160; PSQ and PRQ: low<92, moderate 92–100, high >100. In our sample, the Cronbach's α coefficients for the ORQ, PSQ and PRQ were 0.88, 0.86 and 0.91, respectively.

### Measurement of job burnout

Job burnout was measured by the Chinese version of the Maslach Burnout Inventory Human Services Survey (MBI-HSS), which consisted of 19 test items divided into three dimensions:^5^ ^7^ ^21^ 7 items of EE, 5 items of DEP and 7 items of reduced sense of PA. The first dimension (EE) describes feelings in a general sense, DEP is associated with behaviour and PA involves cognition and feelings affecting self-efficacy.^22^ Some item statements are reversed. Items were scored on a seven-point Likert scale ranging from 1 (strongly disagree) to 7 (strongly agree). The scores for each dimension were computed separately. Scores of EE and DEP >66.7%, together with PA scores <33.3%, were used to code low and high scores. A higher burnout level is predicted by higher scores for the EE and DEP subscales and by lower scores for the PA scale. To better describe burnout states and to more readily identify burnout-associated risk factors, a weighted burnout score was introduced. Structural analysis indicates that exhaustion has the most consistent relationship with burnout; therefore, the equation used for calculations was burnout=0.4×EE+0.3×DEP+0.3×PA. The burnout score was classified into three categories: no burnout (total score from 1 to 2.49), mild burnout (2.50–4.49) and severe burnout (4.50–7). Rural-to-urban migrant workers with mild or severe burnout were defined as ‘burnout cases’.^23^ The MBI-HSS has previously demonstrated that it has high validity and reliability among Chinese medical professionals.^24^ In our current study, the Cronbach's α for EE, DEP and PA was 0.70, 0.77 and 0.74, respectively.

### Additional questions

A questionnaire composed of demographic variables such age, gender, marital status, education level, behaviour customs including smoking, drinking and physical exercise and job-related data such as type of workplace and years of practice was developed for the purpose of the study.

### Statistical analysis

The data were analysed using SPSS for Windows V.19.0 (SPSS, USA). All statistical tests were two-sided, and a p value<0.05 was considered statistically significant. The independent-sample t-test or one-way analysis of variance (ANOVA) was used to compare the means of the MBI-HSS scores in the demographics, behaviour customs and job-related data. Pearson's correlation coefficients were used to examine the correlations among the study variables. Hierarchical linear regression analyses were performed to examine associations between occupational stress and MBI-HSS scores. In the first step of the hierarchical linear regression analyses, the control variables were added into the model. According to the independent-sample t-test and one-way ANOVA analysis, variations such as age, gender, marital status, educational level, smoking, drinking, physical training, hobbies, type of workplace, years of practice, working hours per day and dull or repetitive work were included in the model as potential confounders. In the second step, dimensions of OIS-R were added. Variances of MBI-HSS scores explained by occupational stress were examined by ΔR^2^. A non-conditional logistic regression model was applied to estimate the degree of association between each dimension of occupational stress and the MBI-HSS. The univariate model was first used to identify the potential risk factors, and then the multivariable model was applied to confirm the identified associations.

## Results

### Participant characteristics

Of the 3806 respondents in the study, the average age was 31.35±7.60 years, with 22.04% being under the age of 25 years, 64.69% in 25–40 years old and 13.27% over 40 years old. Over half of participants (52.60%) were men, and 66.55% of the respondents were married. Furthermore, 13.56% of participants had a junior college degree or greater. Most of participants had good behaviour customs: approximately three-quarters (75.60%) avoided liquor and smoking, 72.75% had never smoked cigarettes, 46.24% engaged in physical exercise weekly and 72.77% had an avocation. Among all respondents, 77.04% worked during the day, 41.51% had worked for 5 years or less, 62.61% worked between 8 and 10 hours per day, 61.56% worked monotonously and 62.09% vented their troubles when they faced work pressure.

### Factors associated with job burnout

Table 1 shows differences in burnout subscales according to the respondent's demographics, behaviour customs and job-related characteristics. Marital status, education level, physical exercise, avocation, workplace type, working hours per day and monotonous work were all associated with an EE score. Rural-to-urban migrant workers who were widowed/divorced had higher EE scores than those who were single or married (p<0.05). Rural-to-urban migrant workers with a junior school degree or below had higher EE scores than the other participants (p<0.05). Rural-to-urban migrant workers who engaged in physical exercise every week and had an avocation had lower EE scores than the other respondents (p<0.05). Rural-to-urban migrant workers who worked during the day had lower EE scores compared to shift workers (p<0.05). Rural-to-urban migrant workers with monotonous work who worked ≥10 hours per day had higher EE scores than other participants (p<0.05).

**Table 1:** Univariate analysis of MBI-HSS scores according to demographic, behaviour custom and job-related characteristics

		Emotional exhaustion		Depersonalisation		Personal accomplishment	
Variable	n	Mean±SE	p Value	Mean±SE	p Value	Mean±SE	p Value
Gender							
Male	2017	28.05±5.41	0.870	17.34±3.96	0.049	32.84±6.42	0.000
Female	1789	28.02±5.38		17.09±3.85		31.83±5.93	
Age							
<25	839	27.97±5.33	0.502	17.22±3.81	0.000	32.33±6.08	0.157
25–40	2462	28.11±5.43		17.42±3.91		32.27±6.37	
>40	505	27.82±5.40		16.31±3.96		32.86±5.62	
Marital status							
Single	1216	28.00±5.39	0.034	17.44±3.74	0.012	32.30±6.19	0.061
Married	2533	28.01±5.37		17.11±3.98		32.44±6.22	
Widowed/divorced	57	29.88±6.17		18.11±4.05		30.51±6.26	
Education level							
Junior school or less	1466	28.38±5.57	0.009	17.10±4.01	0.002	32.21±6.15	0.205
High school	1824	27.82±5.37		17.17±3.91		32.55±6.34	
Junior college or more	516	27.83±4.92		17.78±3.55		32.14±5.96	
Smoking							
Yes	1037	28.06±5.34	0.893	17.47±3.97	0.020	32.75±6.65	0.024
No	2769	28.03±5.41		17.14±3.89		32.22±6.04	
Drinking							
Yes	929	28.29±5.38	0.101	17.74±3.97	0.000	32.56±6.54	0.282
No	2877	27.96±5.40		17.06±3.88		32.30±6.10	
Physical exercise							
Yes	1760	27.32±5.33	0.000	17.05±4.21	0.011	32.92±6.98	0.000
No	2046	28.66±5.37		17.38±3.64		31.89±5.43	
Avocation							
Yes	2770	27.67±5.43	0.000	17.00±3.95	0.000	32.70±6.45	0.000
No	1036	29.03±5.15		17.83±3.75		31.48±5.46	
Workplace type							
Fixed	2932	26.94±4.98	0.000	16.53±3.72	0.000	33.68±5.72	0.000
Shift	874	31.72±5.12		19.57±3.63		28.29±6.08	
Years of practice (year)							
≤5	1580	27.80±5.34	0.059	16.91±3.92	0.000	32.57±5.95	0.175
6–10	1043	28.14±5.20		17.51±3.73		32.13±6.24	
>10	1183	28.04±5.40		17.40±4.03		32.30±6.53	
Working hours per day (hours)							
<8	610	27.46±5.54	0.000	16.85±4.00	0.008	32.14±6.57	0.001
8–10	2383	27.95±5.34		17.37±3.92		32.16±6.21	
≥10	813	28.74±5.40		17.10±3.79		33.08±5.91	
Monotonous work							
Yes	2343	28.62±5.54	0.000	17.46±3.85	0.000	32.01±6.10	0.000
No	1463	27.10±5.02		16.85±3.98		32.93±6.36	
Whether troubles are discussed when facing work pressure							
Yes	2363	27.91±5.35	0.063	17.28±4.02	0.275	32.32±6.49	0.585
No	1443	28.25±5.46		17.14±3.73		32.43±5.74	

MBI-HSS, Maslach Burnout Inventory Human Services Survey.

In the dimension of DEP, the mean differences regarding venting one's troubles when faced with work pressure were not statistically significant. Female migrant workers and those aged above 40 years had lower DEP scores compared to other participants (p<0.05). Rural-to-urban migrant workers with a junior school degree or below and who were widowed/divorced had higher DEP scores than the other respondents (p<0.05). Rural-to-urban migrant workers who had good behaviour customs, worked during the day, had been working ≤5 years, worked<8 hours per day and whose work was not repetitive had lower DEP scores compared to the other respondents (p<0.05).

In the case of PA, the mean differences in gender, smoking, physical exercise, avocation, workplace type, working hours per day and monotonous work were statistically significant. Male migrant labourers had higher PA scores than female migrant labourers (p<0.05). Rural-to-urban migrant workers who smoked, engaged in physical exercise weekly and had an avocation had higher PA scores than the other respondents (p<0.05). Rural-to-urban migrant workers who worked during the day had higher PA scores compared to shift workers (p<0.05). Rural-to-urban migrant workers who worked ≥10 hours per day and whose work was not repetitive had higher PA scores than the other participants (p<0.05).

### Correlation between study variables

Table 2 lists the correlations among the MBI-HSS, Occupational Role (OR), Personal Strain (PS) and Personal Resources (PR) variables. Two dimensions of job burnout (EE and DEP) were positively correlated with every item of OR and PS and negatively correlated with PR. PA was positively correlated with two items of OR (responsibility and physical environment) and two items of PS (psychological strain and interpersonal strain) and PR, but was negatively correlated with the other items of OR and one item of PS (vocational strain). There was no significant correlation between PA and physical strain.

**Table 2:** Correlation coefficients among dimensions of MBI-HSS scores and OSI-R

Variable	Emotional exhaustion	Depersonalisation	Personal accomplishment
Occupational role			
Role overload	0.281**	0.284**	−0.071**
Role insufficiency	0.323**	0.227**	−0.213**
Role ambiguity	0.116**	0.363**	−0.294**
Role boundary	0.194**	0.386**	−0.155**
Responsibility	0.038*	0.116**	0.145**
Physical environment	0.295**	0.130**	0.055**
Personal strain			
Vocational strain	0.118**	0.141**	−0.038*
Psychological strain	0.195**	0.155**	0.043**
Interpersonal strain	0.109**	0.092**	0.111**
Physical strain	0.205**	0.208**	0.012
Personal resources			
Recreation	−0.104**	−0.052**	0.196**
Self-care	−0.138**	−0.088**	0.232**
Social support	−0.169**	−0.280**	0.303**
Rational coping	−0.127**	−0.203**	0.244**

*p<0.05; **p<0.01.

MBI-HSS, Maslach Burnout Inventory Human Services Survey; OSI-R, Occupational Stress Inventory—revised edition.

### The relationship between occupational stress and job burnout

Table 3 presents relationship between occupational stress and job burnout in the hierarchical linear regression analyses. After controlling potential confounders listed in tables 1 and 2, a positive association of EE with role overload, role insufficiency, role boundary, physical environment, psychological strain and physical strain was observed. However, EE was significantly reversely associated with role ambiguity, responsibility and vocational strain. Role overload, role ambiguity, role boundary, interpersonal strain and recreation positively predicted DEP, whereas physical environment and social support were negatively associated with DEP. A low level of PA was associated with high role boundary, high role insufficiency, low responsibility, low social support, low physical environment, low self-care and low interpersonal strain (in descending order of standardised estimates). The analysis showed that demographics, behaviour customs and job-related variables explained 17.6%, 13.4% and 14.6% of the variance in EE, DEP and PA, respectively. Occupational stress was responsible for 14.1%, 15.7% and 11.6% of the variance in EE, DEP and sense of PA, respectively.

**Table 3:** Hierarchical linear regression analyses of factors associated with MBI-HSS scores

	Emotional exhaustion		Depersonalisation		Personal accomplishment	
Variable	Step 1 (β)	Step 2 (β)†	Step 1 (β)	Step 2 (β)†	Step 1 (β)	Step 2 (β)†
Gender	−0.094	−0.193	−0.067	−0.073	−0.862**	−0.957**
Age	−0.182	−0.011	−0.474**	−0.359**	0.334	0.220
Marital status	0.083	0.081	0.177	−0.095	−0.198	0.089
Education level	−0.175	−0.322**	0.273**	−0.012	0.009	0.092
Smoking	0.082	0.135	−0.031	0.155	−0.113	0.045
Drinking	−0.399	0.245	−0.587**	−0.121	0.297	0.064
Physical exercise	0.998**	0.579**	0.153	0.363**	−0.639*	−0.270
Avocation	0.574**	0.360*	0.628**	0.216	−0.582*	−0.013
Workplace type	4.622**	3.910**	2.979**	2.428**	−5.117**	−4.324**
Years of practice	0.362**	0.292**	0.414**	0.310**	−0.3576**	−0.400**
Working hours per day	0.560**	0.237	0.096	0.185*	0.509**	0.111
Monotonous work	−1.293**	−0.818**	−0.421**	−0.257*	0.724**	0.551**
Role overload		0.167**		0.045**		0.009
Role insufficiency		0.197**		0.017		0.006
Role ambiguity		−0.144**		0.070**		−0.106**
Role boundary		0.082**		0.149**		−0.151**
Responsibility		−0.052**		−0.001		0.104**
Physical environment		0.073**		−0.025**		0.072**
Vocational strain		−0.087**		−0.013		−0.040
Psychological strain		0.096**		0.003		0.005
Interpersonal strain		−0.011		0.023*		0.047*
Physical strain		0.055**		0.015		0.016
Recreation		0.003		0.028**		0.014
Self-care		−0.026		0.018		0.061**
Social support		−0.018		−0.072**		0.099**
Rational coping		0.009		0.001		0.13
R^2^	0.176**	0.317**	0.134**	0.291**	0.146**	0.262**
ΔR^2^	0.176**	0.141**	0.134**	0.157**	0.146**	0.116**

*p<0.05; **p<0.01. †Step 2 (β) adjusted for gender, age, marital status, education level, smoking, drinking, physical exercise, avocation, workplace type, years of practice, working hours per day, monotonous work, role overload, role insufficiency, role ambiguity, role boundary, responsibility, physical environment, vocational strain, psychological strain, interpersonal strain, physical strain, recreation, self-care, social support and rational coping.

MBI-HSS, Maslach Burnout Inventory Human Services Survey.

In the univariate logistic regression, job strain/personal strain/personal resources are associated with the three dimensions of burnout. The results of the multivariable logistic regression in table 4 were adjusted for rural-to-urban workers' characteristics and occupational stress, including gender, age, marital status, education level, smoking, drinking, physical exercise, avocation, workplace type, years of practice, working hours per day, monotonous work, job strain, personal strain and personal resource. Exposure to low job strain was associated with prevented high EE, high DEP and low sense of PA. Migrant workers with low job strain (OR=0.339, 95% CI 0.251 to 0.458) had less EE than rural-to-urban migrant workers with high job strain. Respondents with low job strain (OR=0.418, 95% CI 0.325 to 0.538) and moderate job strain (OR=0.287, 95% CI 0.242 to 0.341) had lower DEP than respondents with high job strain. Additionally, participants with low job strain (OR=0.768, 95% CI 0.604 to 0.977) and moderate job strain (OR=0.450, 95% CI 0.379 to 0.534) had greater senses of PA than migrant workers with high job strain. However, exposure to low personal strain was associated with prevented high EE and high DEP. Rural-to-urban migrant workers with low personal strain (OR=0.588, 95% CI 0.487 to 0.709) and moderate personal strain (OR=0.782, 95% CI 0.639 to 0.956) had less EE than respondents with high personal strain. Respondents with moderate personal strain (OR=0.759, 95% CI 0.610 to 0.944) had less DEP than respondents with high personal strain. Moreover, exposure to low personal resources increased the risk of high EE, high DEP and a low sense of PA. Rural-to-urban migrant with low and moderate personal resources had 1.353 (95% CI 1.002 to 1.828) times and 2.046 (95% CI 1.506 to 2.780) times risk of presenting EE, respectively, compared to rural-to-urban migrant workers with high personal resources. Respondents with low and moderate personal resources had 2.480 (95% CI 1.840 to 3.343) times and 1.889 (95% CI 1.376 to 2.592) times risk of presenting DEP, respectively, compared to rural-to-urban migrant workers with high personal resources. Additionally, participants with low and moderate personal resources had 3.944 (95% CI 2.935 to 5.299) times and 4.739 times risk of presenting low PA, respectively, compared to migrant workers with high job strain.

**Table 4:** ORs of job burnout by job strain, personal strain and personal resources

Variable	Emotional exhaustion OR (95% CI)†	Depersonalisation OR (95% CI)†	Personal accomplishment OR (95% CI)†	Burnout OR (95% CI)†
Job strain				
Low	0.339 (0.251 to 0.458)**	0.418 (0.325 to 0.538)**	0.768(0.604 to 0.977)*	0.025 (0.007 to 0.082)**
Moderate	0.950 (0.806 to 1.120)	0.287 (0.242 to 0.341)**	0.450 (0.379 to 0.534)**	0.079 (0.024 to 0.256)**
High	1.000	1.000	1.000	1.000
Personal strain				
Low	0.588 (0.487 to 0.709)**	0.841 (0.694 to 1.020)	1.128 (0.935 to 1.361)	0.585 (0.366 to 0.935)*
Moderate	0.782 (0.639 to 0.956)**	0.759 (0.610 to 0.944)*	0.970 (0.785 to 1.199)	0.630 (0.377 to 1.050)
High	1.000	1.000	1.000	1.000
Personal resources				
Low	1.353 (1.002 to 1.828)*	2.480 (1.840 to 3.343)**	3.944 (2.935 to 5.299)**	5.502 (0.757 to 39.995)
Moderate	2.046 (1.506 to 2.780)**	1.889 (1.376 to 2.592)**	4.739 (3.448 to 6.514)**	5.473 (0.747 to 39.500)
High	1.000	1.000	1.000	1.000

*p<0.05; **p<0.01. †Adjusted OR (95% CI), adjusted for gender, age, marital status, education level, smoking, drinking, physical exercise, avocation, workplace type, years of practice, working hours per day, monotonous work, job strain, personal strain and personal resources.

To further explain the relationship between occupational stress and burnout, we also calculated total scores for burnout. The burnout cases included respondents with mild and severe burnout. The results of the univariate logistic regression showed that the three dimensions of burnout are associated with job strain/personal strain/personal resources. After controlling the confounders, table 4 indicates that no association was observed between personal resources and burnout. Exposure to low (OR=0.025, 95% CI 0.007 to 0.082)/moderate job strain (OR=0.079, 95% CI 0.024 to 0.256) and low personal strain (OR=0.585, 95% CI 0.366 to 0.935) was associated with decreased burnout.

## Discussion

In this study, we examined the relationship between occupational stress and job burnout among rural-to-urban migrant workers in China. The findings indicated that burnout was significantly associated with occupational stress in this working population. Reducing occupational stress could be an important strategy to prevent job burnout among migrant workers.

Demographic characteristics including age, gender, education level and marital status might be factors influencing the three dimensions of burnout. In our study, men had more DEP than women, which was consistent with previous studies.^25^ Male migrant workers had higher levels of PA than female migrant workers, probably because men take on few household responsibilities and tasks in dual-career family life.^14^ Migrant workers between 25 and 40 years of age had a higher level of DEP. People in this age group make up the core task force, which can be assigned more tasks, and the greater burden can leave people feeling apathetic. Our findings that widowed/divorced migrant workers had more EE and DEP than single and married workers are supported by other studies.^14^ ^26^ This might be related to the social support provided by a partner. Education level had significant effects on the EE and DEP of migrant workers. Rural-to-urban migrant workers with a higher education level had low levels of burnout. This is probably because migrant workers with lower education accept unskilled jobs.

Behaviour customs such as smoking, drinking, physical exercise and avocation had significant effects on burnout. Rural-to-urban migrant workers with good behaviour customs had a lower level of burnout. This is probably because respondents with good behaviour customs were able to cope more easily with the strain faced at work. Considering the burnout associated with job-related characteristics, burnout varied significantly based on whether or not the respondents worked overnight, whether or not they worked monotonously, the number of years of practice and the number of hours worked per day. A similar result was observed in previous studies,^15^ ^27^ ^28^ in which high burnout was related to heavy workload and job-related stress.

Occupational stress plays an important role in the burnout of migrant workers. Our study showed that the three dimensions of occupational stress were correlated with the three dimensions of job burnout. Rural-to-urban migrant workers who experienced high role overload, high role boundary, low responsibility, high psychological strain and high physical strain had a higher risk of developing high EE, DEP and a low sense of PA. Migrant workers with high role overload and role boundary spent too much time and energy completing their performance goals; thus, they would lack the time to relieve themselves of the competitive pressures they faced. Furthermore, rural-to-urban migrant workers with high psychological and physical strain felt continuously nervous. These factors may contribute to their high EE and DEP as well as their low level of PA. Previous studies suggested that social support improved the ability to manage stress and was effective in reducing burnout.^29^ ^30^ Our hierarchical linear regression analysis showed that those with low social support had a higher risk of developing high DEP and a low sense of PA, probably because social support may buffer the effects of stress.^31^ ^32^ Our current study indicated that job strain, personal strain and personal resources were another predictor of job burnout. High job strain was significantly associated with the three dimensions of burnout and the total job burnout score. Rural-to-urban migrant workers work for long hours and are overloaded, which may lead to physical illness and mental problems. Meanwhile, the pessimism with regard to facing work pressure and the ability to vent work stress may lead to anxiety and depression. The effects on physical and mental health can easily lead to burnout. Our data suggested that the low personal resource was a risk factor for the three dimensions of burnout. This is related to the fact that such individuals rarely engage in recreational activities and lack social support.

### Limitations of this study

Our study is a cross-sectional survey and could not provide a causal relationship for the findings of the association between occupational stress and job burnout. We collected the data through the participants' self-reporting; thus there may be a potential bias due to the self-report methods. The study population was selected from the two towns only, and the generalisation of the findings may be limited. All findings obtained in the present study must be confirmed in future prospective studies.

## Conclusion

This study found that occupational stress was associated with job burnout among migrant workers. Strategies to reduce occupational stress as well as maintain an appropriate quantity and quality of work need to be developed and should be crucial to preventing job burnout of migrant workers in China.

## References

[1] Zeng Y Review of work-related stress in mainland Chinese nurses. Nurs Health Sci 2009;11:90–7. 10.1111/j.1442-2018.2008.00417.x 19298314

[2] Makin PJ, Rout U, Cooper CL Job satisfaction and occupational stress among general practitioners—a pilot study. J R Coll Gen Pract 1988;38:303–6. 3255825 PMC1711473

[3] Tsai FJ, Huang WL, Chan CC Occupational stress and burnout of lawyers. J Occup Health 2009;51:443–50. 10.1539/joh.L8179 19590156

[4] Montgomery A, Panagopolous E, Benos A Work–family interference as a mediator between job demands and job burnout among doctors. Stress Health 2006;22:203–12. 10.1002/smi.1104

[5] Maslach C, Jackson S, Leiter M Maslach Burnout Inventory. 3rd edn Palo Alto, CA: Consulting Psychologists Press, 1996.

[6] Honkonen T, Ahola K, Pertovaara M The association between burnout and physical illness in the general population—results from the Finnish Health 2000 Study. J Psychosom Res 2006;61:59–66. 10.1016/j.jpsychores.2005.10.002 16813846

[7] Maslach C, Schaufeli WB, Leiter MP Job burnout. Annu Rev Psychol 2001;52:397–422. 10.1146/annurev.psych.52.1.397 11148311

[8] Lindwall M, Gerber M, Jonsdottir IH The relationships of change in physical activity with change in depression, anxiety, and burnout: a longitudinal study of Swedish healthcare workers. Health Psychol 2014;33:1309–18. 10.1037/a0034402 24245832

[9] Gorji M, Vaziri S The survey job burnout status and its relation with the performance of the employees (case study: bank). Int Proc Econ Dev Res 2011;14:219–24.

[10] Han L, Shi L, Lu L Work ability of Chinese migrant workers: the influence of migration characteristics. BMC Public Health 2014;14:353 10.1186/1471-2458-14-353 24725332 PMC4020317

[11] National Bureau of Statistics of China. National report on migrant workers of China, 2012 http://www.gov.cn/gzdt/2013-05/27/content_2411923.htm.

[12] National Bureau of Statistics of China. National report on migrant workers of China, 2011 http://www.stats.gov.cn/ztjc/ztfx/fxbg/201204/t20120427_16154.html.

[13] Zhang X, Wang Z, Li T The current status of occupational health in China. Environ Health Prev Med 2010;15:263–70. 10.1007/s12199-010-0145-2 21432554 PMC2921040

[14] Wang Y, Ramos A, Wu H Relationship between occupational stress and burnout among Chinese teachers: a cross-sectional survey in Liaoning, China. Int Arch Occup Environ Health 2015;88:589–97. 10.1007/s00420-014-0987-9 25256806

[15] Jin MU, Jeong SH, Kim EK Burnout and its related factors in Korean dentists. Int Dent J 2015;65:22–31. 10.1111/idj.12142 25412905 PMC9376508

[16] Tijdink JK, Vergouwen AC, Smulders YM Emotional exhaustion and burnout among medical professors; a nationwide survey. BMC Med Educ 2014;14:183 10.1186/1472-6920-14-183 25189761 PMC4167137

[17] Escriba-Aguir V, Martin-Baena D, Perez-Hoyos S Psychosocial work environment and burnout among emergency medical and nursing staff. Int Arch Occup Environ Health 2006;80:127–33. 10.1007/s00420-006-0110-y 16710712

[18] Wu S, Zhu W, Wang Z Relationship between burnout and occupational stress among nurses in China. J Adv Nurs 2007;59:233–9. 10.1111/j.1365-2648.2007.04301.x 17590211

[19] Osipow S Occupational Stress Inventory Revised Edition. Odessa: Psychological Assessment Resources, 1998.

[20] Hayne AN, Gerhardt C, Davis J Filipino nurses in the United States: recruitment, retention, occupational stress, and job satisfaction. J Transcult Nurs 2009;20:313–22. 10.1177/1043659609334927 19372539

[21] Boles JS, Dean DH, Ricks JM The dimensionality of the Maslach Burnout inventory across small business owners and educators. J Voc Beh 2000;56:12–34. 10.1006/jvbe.1999.1689

[22] Nowakowska-Domagala K, Jablkowska-Gorecka K, Kostrzanowska-Jarmakowska L The interrelationships of coping styles and professional burnout among physiotherapists: a cross-sectional study. Medicine (Baltimore) 2015;94:e906 10.1097/MD.0000000000000906 26091455 PMC4616538

[23] Wang Z, Xie Z, Dai J Physician burnout and its associated factors: a cross-sectional study in Shanghai. J Occup Health 2014;56:73–83. 10.1539/joh.13-0108-OA 24430838

[24] Wu S, Li H, Zhu W Effect of work stressors, personal strain, and coping resources on burnout in Chinese medical professionals: a structural equation model. Ind Health 2012;50:279–87. 22673361 10.2486/indhealth.ms1250

[25] Lin QH, Jiang CQ, Lam TH The relationship between occupational stress, burnout, and turnover intention among managerial staff from a Sino-Japanese joint venture in Guangzhou, China. J Occup Health 2013;55:458–67. 10.1539/joh.12-0287-OA 24025859

[26] Bauer J, Stamm A, Virnich K Correlation between burnout syndrome and psychological and psychosomatic symptoms among teachers. Int Arch Occup Environ Health 2006;79:199–204. 10.1007/s00420-005-0050-y 16258752

[27] Linzer M, Visser MR, Oort FJ Predicting and preventing physician burnout: results from the United States and the Netherlands. Am J Med 2001;111:170–5. 10.1016/S0002-9343(01)00814-2 11498074

[28] Goehring C, Bouvier Gallacchi M, Kunzi B Psychosocial and professional characteristics of burnout in Swiss primary care practitioners: a cross-sectional survey. Swiss Med Wkly 2005;135:101–8. doi:2005/07/smw-10841 15832226 10.4414/smw.2005.10841

[29] Prag PW Stress, burnout, and social support: a review and call for research. Air Med J 2003;22:18–22. 10.1016/S1067-991X(03)00021-X 14671770

[30] Baruch-Feldman C, Brondolo E, Ben-Dayan D Sources of social support and burnout, job satisfaction, and productivity. J Occup Health Psychol 2002;7:84–93. 10.1037/1076-8998.7.1.84 11827236

[31] Ward L Mental health nursing and stress: maintaining balance. Int J Ment Health Nurs 2011;20:77–85. 10.1111/j.1447-0349.2010.00715.x 21371222

[32] Hughes H, Umeh K Work stress differentials between psychiatric and general nurses. Br J Nurs 2005;14:802–8. 10.12968/bjon.2005.14.15.18597 16116406

